# Vitality and Immunity

**Published:** 1903-01

**Authors:** Carl Schulin

**Affiliations:** Billings, Mont.


### THE JOURNAL

OF

### COMPARATIVE MEDICINE AND VETERINARY ARCHIVES.

VOL. XXIV. *JANUARY, 1903.* No. 1.

### VITALITY AND IMMUNITY.

BY CARL SCHULIN, M.D.,

BILLINGS, MONT.

PREVENTIVE medicine is the art of preserving or prolonging life by the prevention of disease. As in the case of all other medical science, during the second half of the last century, a radical change ensued through discoveries following the improvement of the compound microscope.

Fifty years ago it was generally assumed that after death flesh decays from intrinsic causes. The life of the individual was considered a complicated process the nature of which involved a termination by death, and it was taken for granted that, as soon as life was extinct, the body disintegrated. The only feature of immortality which was conceded to the body was its capability of producing other bodies of the same kind. It was admitted that the number of generations was unlimited and could be terminated only by external causes. The individual, however, was pronounced to be absolutely and invariably doomed to die, not only from external causes, but as a natural sequence of construction.

Pasteur and his school have since demonstrated that flesh does not decay from intrinsic causes, but through the influence of invading germs, and can be preserved by their exclusion. An entirely new branch of the medical science, namely, bacteriology, was developed, with the result that in a large number of diseases it was demonstrated that they originate from the invasion of germs and can be prevented by guarding against them.

While Pasteur successfully exposed the rôle of germs, another great French savant did pioneer work in the metabolism of the body. Brown-Séquard, the discoverer of the inner secretions, first conceived the idea that senility might not be a natural consequence of the normal metabolism. He pronounced it a disease which is caused by the exhaustion and might be treated successfully by the replenishment of certain products of animal life. He, too, created a new branch of the medical science, namely, organotherapy. It is young, but growing rapidly, and bids fair to become the nucleus of therapeutics.

Preventive medicine is as old as history. Its scientific foundation, however, was created only quite recently by Pasteur and Brown-Séquard. Before their time it consisted only of common-sense regulations how to keep the body clean and the food pure, to isolate contagious diseases, and of attempts at restricting the excessive use of intoxicating drinks. It was not so much in the hands of medical men as of jurists and clergymen. In ancient times the clergy controlled more or less all branches of medicine and used their influence for the enforcement of sanitary measures. Naturally, they took an especially great interest in the promotion of temperance, as they still do to-day.

Concerning the efforts of the clergy to wean the world from intoxicating drinks the writer has observed a strange coincidence. The ancients had only fermented drinks, namely, wine and beer. As far back as the days of Pharaoh, the Egyptians shook the dice for a glass of beer. All the ancient nations, at one time or another, found it necessary to regulate by law the sale of wine and beer, but they never thought of prohibiting drinking absolutely. The first man who conceived such an idea was Mohammed. He knew only of wine and forbade his followers to drink it. The Arabians, however, were good chemists, and soon discovered that by distillation something could be made out of wine which, though not wine, had the same effects. Thus the invention of distilled liquors which has proved a curse to humanity, was the direct consequence of the first prohibition law.

The first physician who made a genuine medical discovery in the line of preventive medicine was Edward Jenner. In his work, *An Inquiry into the Causes and Effects of the Variolæ Vaccinæ*, which he published in 1798, he furnished conclusive proof that inoculation with cowpox protects against smallpox. Long before Jenner's day it was known that a similar result could be obtained by inoculation from a smallpox pustule in an advanced stage. This method, however, was prevented from coming into general use by the danger it involved of spreading the very disease which it was intended to guard against. Jenner made his discovery accidentally. He could not give a theoretical explanation of it. He only recognized its enormous practical value and made it the object of his life to introduce it in larger circles.

Only to-day is the theoretical explanation of Jenner's discovery dawning upon our minds. The puzzle is far from solved, but our knowledge is growing daily. The start was made by Pasteur. His work led, in England, through Lister, to discoveries which revolutionized surgery; and in Germany, through Koch and his school, to the invention of entirely new methods of treating diseases specifically.

It would be beyond the narrow limits of this paper to give even the shortest synopsis of the present status of bacteriology. The writer only wishes to reproduce before the reader's mind a few well-known facts in the light of preventive medicine. Old facts in a new light assume a new aspect.

The line between preventive and curative measures is naturally not very closely drawn. This shows itself in the different methods adopted in dealing with germs. They are:

1. Methods of preventing their entrance into the body. This, of course, is one of the main branches of preventive medicine.

2. Methods of destroying the germs in the body, or of expelling them, or of counteracting the effect of their depredation. These measures are more curative than preventive.

3. Methods of placing the body in such a condition that germs cannot do any harm. This is accomplished through immunization.

Immunity means capability of exposing one's self to infection with impunity. The word immunity comes from the Latin word immunis, which meant a man who was excused from meeting the pecuniary and other obligations of a citizen. These obligations were called munia. Only those who met them were full members of the communitas. The immunis was only an honorary member, free from the obligations and void of the rights of citizenship. He was, in ancient Rome, as well protected against the collector of taxes, as the immune colored soldier, in our war with Spain, was protected against yellow fever.

Immunity against a certain disease may be congenital or acquired. It may be absolute or partial, so that only a mild case can be contracted. The susceptibility of different animals to a certain disease vary greatly. For instance, mice and rabbits are the most susceptible to streptococcus pyogenes; dogs and cats in a lesser degree. Still less susceptible are sheep and goats; and the horse and ass the least of all. Still, there is no animal absolutely immune against streptococcus pyogenes, nor can complete immunity against it be acquired. A man who has had a case of smallpox can hardly contract it again. An infection with streptococcus pyogenes, however, does not protect against another infection of the same kind.

The most common germs are those which produce inflammation and putrefaction. Other germs, for instance the typhoid bacillus, are capable of producing inflammation. As a rule, however, it is caused by streptococcus and staphylococcus pyogenes. If the typhoid bacillus causes an abscess, these two cocci will soon put in their appearance. They follow the typhoid bacillus, but the typhoid bacillus does not follow them. And wherever the cocci or any other germs have been active for any length of time, the germs of putrefaction will also be there.

As soon as the cocci invade the body, the leucocytes rush together from all sides. Some try to kill the intruders. Others liquefy the tissues around their colony and try to make an opening through which the enemy can be ejected. Others again build a wall around the cavity which their brethren have made, to protect the balance of the body against further invasion and to furnish the material for repair of the damage done.

The success of the cocci depends upon their virulence, which is very variable and in its turn depends upon the conditions under which they had been living prior to attacking the body. If the virulence be great enough, the cocci will conquer the fighting, the liquefying, and even the wall building leucocytes and spread all over the body. They will eventually cause death under the symptoms of what is known as pyæmia. If the conditions are favorable to the leucocytes, an abscess will be formed under the old-time symptoms of tumor, rubor, calor, dolor, and in the end by its opening with or without the surgeon's knife, the incident will close with a discharge of pus *bonum et laudabile.*

If, however, for some reason the pus cavity cannot be evacuated; if neither the leucocytes are strong enough to do their part, nor the cocci able to break through, then both will fall victims to the putrefactive bacteria which at this time put in their appearance. The pus *bonum et laudabile* will change to pus *malignum.* It becomes a foul-smelling mass in which poisonous substances develop. The result will be an incapsulated abscess. Its cavity is lined with a so-called pyogenic membrane. The principal feature is the absence of oxygen. The incapsulated abscess cavity resembles an old tomb-vault. Its lining membrane shows no healthy red granulations from which the pus could be easily removed. It has a bluish and dirty greyish appearance and the cheesy pus is firmly attached to the granulations by a felt-like mass of coagulated fibrin. This is also in a putrid condition. The outside of the pyogenic membrane looks dark bluish and, if you cut into it, dark blood escapes which the people of all times called "bad blood." The cavity may be connected with the outside world by a tortuous canal, a so-called fistula, without otherwise changing its condition.

Reabsorption from such a cavity causes a condition which is known as septicæmia. It may also lead to death from exhaustion and amyloid degeneration of vital organs.

An incapsulated abscess need not be very large. It can be of very small size and even without a cavity. It can be a small nodule of granulating tissue with a slimy or cheesy centre where the intruded bacilli lead a saprophytic life. This is the way in which germs of putrefaction establish themselves in our bodies.

In the same sense, the leucocytes are the soldiers of the body, the lymphatic glands are its military posts. They are regularly garrisoned with leucocytes. Everything that enters into our system, before being admitted to the blood, has to pass through some of these posts. So do the invading germs. The virulent ones will produce suppuration; a so-called bubo. A good many will be destroyed by the leucocytes. Some, however, will succeed in establishing a permanent home right in the headquarters of the enemy; a constant danger, a detriment to the body. They produce what the physicians of former ages called dyscrasia. It occurs especially in children where the thus affected and therefor enlarged lymphatic glands are the principal symptom of scrofulosis.

As we cannot make our patients immune against the pyogenic and putrefactive germs, we protect them against infection of the wound on the operating-table by perfect cleanliness. The air of the operating-room, the surfaces of the bodies of the operator as well as the patients; even the breath of the operator, all contain cocci. Everywhere within reach of our patients the cocci must be killed or removed or prevented from entering the wound.

The condition of the wound thus produced has been improperly called aseptic. The surgeon takes his preventive measures, not against sepsis, but against inflammation. Healthy blood and tissues have a slight antiseptic power. The anaërobic germs of putrefaction cannot withstand the oxygen of the tissues. The tissues, however, have not the power of destroying cocci. The leucocytes must do it! The surgeon, however, wants the leucocytes to attend to the healing of the wound, and he wants only so many to come as there are necessary to do this. He wishes to avoid the rush of leucocytes which is produced by the cocci and might lead to suppuration. Therefore, he keeps the cocci out of the way. The presence of a few germs of putrefaction could do no harm. The tissues would dispose of them quietly and quickly.

Recently it has become customary to say "sterile" instead of "aseptic." Neither is correct. Sterile, in opposition to fertile, means that the ground is in such a condition that nothing can be grown in it. The condition under discussion, however, does not compare with sterile, but rather with fallow ground. On fallow ground growth is prohibited only by the absence of seed. The proper term for the condition of a perfectly clean wound would be "germless" or "free from germs."

The distinction between the different, not only varieties, but even species, of bacteria is not so well defined as one might suppose. In every case different species and varieties are to be found mixed together. The essential types which they are named after are always well represented; but one finds as well many indistinct forms of transition concerning which there is doubt as to which side they belong to.

There is no coccus better characterized than the gonococcus, and it has features which are of special interest in the light of preventive medicine. It has never been found outside of the human body, except on such things as dirty linen, which it reached directly from its regular habitat. Like the typhoid bacillus, it cannot exert its energies on any other but the human body. All animals have absolute congenital immunity against both, but man cannot acquire immunity even against the gonococcus. The typhoid bacillus, having punished its victim long enough, leaves it in a condition of immunity against another infection. The gonococcus may reaffect, no matter how many times a man has already had gonorrhœa. The immunity of animals against both of these germs has in some instances been conquered by pure cultures of germs raised under such conditions as tend to increase their virulence.

Every physician meets once in a while with a case of gonorrhœa which makes him wish that another kind of origin of this disease, besides the only one now recognized, might be discovered. And the possibility of this cannot be absolutely denied. In an otherwise healthy vagina sometimes diplococci are found which so closely resemble the gonococci that the distinction can only be made through certain methods of staining. It has been shown that different varieties of staphylococcus change one to another; why should not some of these non-virulent diplococci, under certain conditions, change to gonococci? The writer believes in evolution, also of germs and heartily endorses the ideas which Lydston expresses in a paper with the title "The Evolutionary Aspect of Infectious Diseases," etc. (*Journal of the American Medical Association*, 1902, Nos. 20 and 21), especially when he advocates the "spontaneous generation of new and virulent properties in hitherto innocuous germs."

(To be continued.)

---

Dr. W. G. Benner, of Doylestown, Pa., who recently entered the Bureau of Animal Industry, has been stationed at Chicago. Ill.

